# The effect of women’s bargaining power within couples on contraceptive use in Cameroon

**DOI:** 10.12688/gatesopenres.13100.1

**Published:** 2020-02-10

**Authors:** Dimitri Tchakounté Tchuimi, Benjamin Fomba Kamga

**Affiliations:** 1 Faculty of Economics and Management, University of Yaounde II, Yaounde, Centre, Po. Box 1792, Cameroon

**Keywords:** Women’s bargaining power, Contraceptive use, Couple, Maternal death, Probit model, Cameroon

## Abstract

**Background:** The prevalence of contraception among married women, evaluated at 23%, is low in Cameroon. Maternal death rates, estimated at 782 deaths per 100,000 live births, are very worrying. The National Strategic Plan for Reproductive, Maternal, Newborn and Child Health (2015–2020) focuses on increasing contraceptive prevalence as a means to reduce maternal death. This paper identifies women’s bargaining power as a factor that may stimulate contraceptive use. The objective of this study is to measure the effect of women's bargaining power within couples on contraceptive use.

**Methods:** The data used come from the Demographic and Health Survey and Multiple Indicators (DHS-MICS) conducted in 2011. Women’s bargaining power within couples is measured by a Woman Bargaining Power Composite Index (WBPCI) built through a multiple correspondence analysis. Descriptive statistics (frequency distribution, cross tabulation, chi-square test) and the probit model were used to analyze the relationship between WBPCI and contraceptive use. Additionally, since the target population for this study is couples in which women were not pregnant, a Heckman probit model was also estimated to address the potential selection bias.

**Results:** The results of the descriptive statistics show that women's bargaining power is higher among women who use contraception than for those who do not. The results of the probit model show that women's bargaining power significantly increases the probability of contraceptive use by 3.4%. In addition, the probability of using contraception also increases with the education of women. The estimation of the Heckman probit model illustrates that the effect of women's bargaining power on the probability of contraceptive use remains virtually unchanged.

**Conclusions:** To reduce high maternal death rates in Cameroon, public health policies should not only focus on the health system itself but should also focus on social policies to empower women in the household.

## Introduction

Improving maternal health is one of the key priorities of public health policies in the world in general, especially in developing countries. Defined in 2000 as the fifth Millennium Development Goal (MDG-5), the reduction of maternal deaths was renewed in 2015 as a target of the third Sustainable Development Goal (SDG-3). For a woman, pregnancy is a period of the life cycle during which her health is particularly vulnerable. In fact, the complications observed throughout this period seem to be the main cause of maternal deaths in developing countries ( Haider *et al.*, 2017). The use of reproductive health care services, particularly in relation to the use of contraception to control fertility and to prevent early and unwanted pregnancies, appears to be an investment to ensure good health for the mother. Thus, access to reproductive health care is an effective weapon for limiting the risk of maternal morbidity and death ( Hou & Ma, 2013).

In developing countries, several factors may be associated with the use of reproductive health care services. The 1994 International Conference on Population and Development (ICPD) in Cairo already emphasized the role of women's economic empowerment. Moreover, as it is recognized that decisions that lead women to use reproductive health care services occur in the sphere of marriage, the household or the family ( Becker, 1996), much literature has suggested that appreciation of women's bargaining power ^1^, while at the same time reducing gender inequalities in the couple, is an important driver for the use of reproductive health care services ( Blanc, 2001; Chapagain, 2006; Haque *et al*., 2012; Kamiya, 2011). It is generally recognized that women's bargaining power over resource allocation and decision-making is assessed through so-called modern factors, such as education, income, possession of property, legislation prohibiting violence against women and the freedom to move about ( Adjiwanou *et al.*, 2018; Allendorf, 2007; Beegle *et al.*, 2001; Habibov *et al.*, 2017). However, the power of women is hardly indissociable from the channel of social and cultural norms on the allocation of resources in the household and the role of women in the organization of the household and the couple; in most cases, these standards restrict women in decision-making both for themselves (decisions about the use of adequate health care, among others) and their children. As a result, since women's traditional values may hinder the positive impact of their modern values, the mechanisms by which women's bargaining power can influence the use of reproductive care appear delicate and ambiguous ( Westeneng & D'Exelle, 2015), drawing our attention to the specificity of the context in which the study is conducted.

In Cameroon, the assessment of the use of reproductive health care is of particular importance since maternal death levels increased considerably between 2004 and 2011, from 669 to 782 deaths per 100,000 births. Current government policy on reproductive health focuses on facilitating access to reproductive care (with a focus on assessing modern contraceptive prevalence) as a means of reducing these high rates of maternal mortality. However, it is becoming increasingly clear that issues related to reproductive health are more of a joint behavior in a couple than an individual choice. Indeed, in Africa, marriage remains the privileged place of procreation ( Noumbissi & Sanderson, 1999) and where the decision to use adequate reproductive health care results from the ability of each partner expressing preferences through negotiation ( Nikiema *et al*., 2008).

This paper aims to evaluate the effect of women's bargaining power within couples on contraceptive use in Cameroon. The contribution of this study is at three main levels. First, information from work linking women's bargaining power in the couple and reproductive health is fairly abundant in countries with a strong tendency to conserve patriarchal cultures. However, this type of investigation is particularly rare in Cameroon, a country not bordering on the prevalence of asymmetrical gender relations and power imbalances between spouses. This study will fill the gap related to this information scarcity. Secondly, this study aims to provide an effective tool for achieving the goal of reducing maternal death rates of the current health policy in the horizon of 2020 ^2^. Thirdly, it incorporates the idea that there is a potential sample selection bias due to the fact that the study focuses on non-pregnant women at the time of survey.

## Methods

### Ethical statement

Permission to use the datasets for this research was obtained prior to the study from the Demographic and Health Surveys (DHS) Program. Ethical approval was not required for this study because it used anonymized DHS data only. The IRB-approved procedures for DHS public-use datasets do not in any way allow respondents, households, or sample communities to be identified.

### Data

The data come from the fourth DHS conducted jointly with the Multiple Indicator Cluster Survey in Cameroon (DHS-MICS) in 2011. This survey was conducted by the National Institute of Statistics (INS) in collaboration with the Ministry of Public Health and financial support from the United States Agency for International Development (USAID), United Nations Population Fund (UNFPA) and UNICEF. As in other developing countries, this survey has collected information on household characteristics, reproductive and sexual health/AIDS, children's health, STD/AIDS knowledge, attitudes and behaviors, and the bargaining power of women in households. Three questionnaires permitted us to collect the data in this survey: the women's questionnaire, the men's questionnaire and the household questionnaire. These different questionnaires generated five modules: the child, woman, man, household and couple modules. As part of this analysis, we will use the couple (monogamy) module within which the answers to the questions were reported by women aged 15 to 49 who reported being married or living in a union with a partner.

In total, a representative sample of 2,973 couples was constituted at the end of the survey. However, since we are interested in the use of contraception at the time of the DHS-MICS survey, the analysis is limited to couples in which women were not pregnant. The data comprised of a total of 461 couples where each woman was pregnant at that time was therefore excluded. Thus, the final sample consists of 2,512 couples.

### Variable measurement

***Dependent variable.*** The dependent variable of this paper is the use of contraception. It is measured by a variable that takes the value 1 if the woman uses a modern or traditional contraceptive method at the time of the survey, and 0 if not. The modern contraceptive methods include the pill, the intra-uterine device (IUD), the injection, the condom, the implant/Norplant, the female condom and sterilization. The traditional methods include periodic abstinence, withdrawal, breastfeeding, and amenorrhea.

***Independent variables***

**The variable of interest, women’s bargaining power**

The transition from the theoretical conception of the woman's bargaining power to her empirical measure is associated with many difficulties since the process of negotiation between the partners within a couple is unobservable ( Beegle *et al.*, 2001; Friedberg & Webb, 2006). Thus, to measure the bargaining power of women, it is essential to resort to proxies ( Doss, 2006). A review of the literature reveals that several dimensions can be used to design it ( Ewerling et al., 2017; Hanmer & Klugman, 2016; Lépine & Strobl, 2013; Mahmud *et al.*, 2012).

In this research, women's bargaining power is apprehended through six specific dimensions: decision-making on key aspects of household life, control over financial resources, economic status/independence in the household, women's attitudes towards husband’s violence (a dimension to understand the contribution of cultural norms to the role of gender justifying the domination of the husband over the women), belonging to community groups/associations and the interruption of the interview by an adult (the husband, another man or a woman). A detailed description of each of these dimensions and their indicators is provided as *Extended data* (Table A1; Tchakounte, 2020). A multiple correspondence analysis (MCA) on the indicators that makes up each of these dimensions has permitted us to generate a Women Bargaining Power Composite Index (WBPCI) used in our investigations to measure the bargaining power of women. The results of this MCA are provided as *Extended data* (Table A2; Tchakounte, 2020) ^3^.

Keeping the continuous values of the WBPCI has the relevance of avoiding ambiguities related to a categorization of this index in various modalities ( Haider *et al.*, 2017). As Richardson (2018) points out, using threshold values to categorize such an index is not appropriate as it is only a matter of value judgments and a subjective approach that sometimes may not reflect the context in which the study is conducted.

However, it should be noted that the WBPCI values obtained after the MCA are negative and positive (between -2.21 and 4.07), which may lead to ambiguities in the interpretation of this index and its likely effect on contraceptive use. Therefore, a normalization of the WBPCI will be made in order to make its values only positive (greater than or equal to zero). Thus, if *Min_WBPCI* is the minimum value of the WBPCI, then this normalization consists in applying the formula: *WBPCI -Min_ WBPCI*.

In addition, Cronbach's alpha coefficient is used to measure the internal consistency of the WBPCI. The closer the value of this coefficient is to 1, the more reliable the constructed index is ( Aiken & West, 1991). Here, the value of Cronbach's alpha obtained is 0.716, which indicates the reliability of the WBPCI.

**Control variables**

Control variables are divided into three categories: socio-demographic, economic and cultural factors, media exposure factors, and intermediate variables of contraception. The first category of factors includes age, education, work status, religion, number of living children, household wealth and place of residence. Two factors of women’s exposure to the media are considered: exposure to television and exposure to radio. For the third category of factors, two intermediate variables of contraception are considered: the desire of the woman not to have an additional child and the husband's approval of family planning. Table 1 highlights the description, measurement and codes of control variables.

**Table 1.** Description, measurement and codes of control variables

Variables	Description and measurement
***Sociodemographic, economic and cultural factors***	
Age of woman	Age (number of years) of the woman at the time of the survey.
Age of partner	Age (number of years) of the partner at the time of the survey.
Education of woman	The highest level of education attained by women measured by four dummy variables: uneducated, primary, secondary and higher level.
Education of partner	The highest level of education attained by partner measured by four dummy variables: uneducated, primary, secondary and higher level.
Status of occupation of husband	Measured by a binary variable taking the value 1 if the husband worked in the 12 months preceding the survey and 0 if not.
Religion of woman	Represents the religious affiliation of the woman measured by three dummy variables: Muslim, Christian and other religions.
Number of living children	Represents the number of living children of the couple.
Household wealth	Represents the household's living standard and measured using the wealth index, which categorizes household into five wealth quintiles: poorest, poor, average, rich and richest ^4^.
Place of residence of household	Measured by a binary variable taking the value 1 if the household lived in a rural area and 0 if they lived in an urban area ^5^.
***Factors of exposure to media***	
Exposure to TV	Number of times the woman watched TV per week and measured by a dummy variable: 1 if at least once per week and 0 if not once.
Exposure to radio	Number of times the woman listened to the radio per week and measured by a dummy variable: 1 if at least once per week and 0 if not once.
***Intermediate variables of contraception***	
Approval of family planning by husband	Measured by a dummy variable taking the value 1 if the husband approved family planning and 0 if not.
Desire of the woman to have no more children	Measured by a dummy variable taking the value 1 if the woman no longer wanted to have children and 0 if not.

### Empirical methods

In addition to descriptive statistics (cross tabulation and chi-square tests), this paper also uses an econometric model. To this end, let us recall that the objective of the research is to measure the effect of women's bargaining power within couples on contraceptive use. Since contraceptive use is a dichotomous variable, a qualitative dependent variable model, and more specifically a probit model, is used.

***Specification of probit model.*** The probit model can be formalized using the following equation:

$$C_{i}^{*}=\beta_{0}+\beta_{1}WBP_{i}+\beta_{2}X_{i}+\varepsilon_{i}\;[1]$$

Where $C_{i}^{*}$ corresponds to the probability that the married woman of couple *i* use one contraceptive method. It’s the latent variable of contraceptive use, *C _i_*, defined as:

$$C_{i}=\{\begin{array}{c} 1\;\text{if}\;{\text{C}}_{\text{i}}^{*}>0\\ 0\;\text{if}\;{\text{C}}_{\text{i}}^{*}\leq 0 \end{array}$$

The probability of using a contraceptive method is expressed as follows:

$$P(C_{i}=1)=P(C_{i}^{*}>0)=P(-\varepsilon_{1i}\leq \beta_{0}+\beta_{1}WBP_{i}+\beta_{2}X_{i})=(\beta_{0}+\beta_{1}WBP_{i}+\beta_{2}X_{i})$$

Where φ is the normal distribution function.

*WBP _i_* corresponds to woman’s bargaining power of couple *i* and is measured by WBPCI. *β* is the coefficient which allows measurement of the effect of woman’s bargaining power on contraceptive use. *X* is the matrix of control variables, and *γ* is the vector of parameters which permits assessment of the effect on contraceptive use of control variables contained into *X*. ε is the error term, independently and identically distributed.

However, it should be noted that to analyze the effect of women's bargaining power on contraceptive use, we restricted the sample to women who were not pregnant at the time of survey and 461 women were excluded. Therefore, to provide a more robust analysis, it is relevant to address the potential selection bias that may exist as a result of the exclusion of pregnant women from the sample.

***Taking into account the selection bias: specification of Heckman probit model.*** Indeed, if the probability of not being pregnant or having a child is correlated with the woman's bargaining power, this would lead to a potential source of sample selection bias. The results obtained from the probit model could then be biased. To produce results in accordance with the potential selection bias, the probit model with sample selection, or Heckman probit model, will be estimated. As specified in the equation [1], the probit model is written as: $C_{i}^{*}=\beta_{0}+\beta_{1}WBP_{i}+\beta_{2}X_{i}+\varepsilon_{i}$ where C is observed only for C* > 0.

The equation of selection is presented as follows:

$$O_{i}=\beta_{0}+\beta_{1}S_{i}+\beta_{2}WBP_{i}+\beta_{3}X_{i}+\vartheta_{i}\;[2]$$

where *O _i_* take the value 1 if the woman wasn’t pregnant at the time of the survey and 0 if not. *S* is an additional exogenous variable of the selection equation, corresponding to the ideal family size of the woman. ε *_i_* ~ *N*(0,1), *_i_* ~ *N*(0,1) and *ρ* = *corr*(ε *_i_*, *_i_*), that is *ε* and are the error terms that follow a standard normal distribution and, the parameter *ρ* corresponds to their correlation. Nevertheless, as highlighted by Van de Ven & Van Pragg (1981), the probit model with sample selection assumes that dependent variable *C* for observation *i* is observed if *O _i_* > 0. Thus, from the equation [2], to be certain of the existence or not of a selection bias, a likelihood ratio test on the null hypothesis *H*_0_: *ρ* = 0 is performed. If this hypothesis is accepted, the error terms are independent and the equations [1] and [2] have no relation. In this case, there is no selection bias. On the other hand, if this hypothesis is refuted, the error terms are linked, which means that the probability of not being pregnant is correlated with the woman's bargaining power. In this case, there is a selection bias and the Heckman probit model would be more appropriate than the probit model.

### Software

The software used to carry out the empirical analyzes of this study was STATA, version 15 ( StataCorp, 2017).

## Results and discussion

### Results of descriptive statistics

The results of the descriptive statistics are divided into two parts: univariate and bivariate statistics.

***Univariate statistics.*** Table 2 highlights the descriptive statistics of the variables of the study. It appears that among the non-pregnant women at the time of the survey, only 23% used one contraceptive method. Overall, women in a union have a low bargaining power within the couple since the average value of WBPCI is 2.301 (knowing that the maximum value is 6.26 and the minimum is 0). Looking at control variables, the average age of married women is 31, and 40 for their partner. Among these women, the most representative level of education is the primary level (41%), followed by the secondary level (29.5%), uneducated (27%) and the higher level (3%). In addition, most of partners have a primary level of education (36%), followed by secondary level (35%), uneducated (17.5%) and a higher level (7%). Almost all partners (99%) had an occupation in the 12 months preceding the survey. The most prevalent religion among married women is Christianity (71%), with 23% of Muslim religion. On average, couples had three living children. Regarding household wealth, 22% of households were poor, 21% very poor, 20% of average wealth, 19% very rich and 18% were rich. In addition, 60% of households lived in rural areas. Regarding media exposure variables, 55.5% of married women watched TV at least once a week and 33.5% listened to the radio at least once a week. Finally, for the intermediate variables of contraception, 33.5% of married women no longer wanted to have children and 36% said that their partner approved family planning.

**Table 2.** Descriptive statistic of variables

Variables	Mean/proportion	Std. Dev.	Min	Max
Contraceptive use	0.229	0.42	0	1
WBPCI	2.301	0.952	0	6.28
Age of woman	31.414	8.522	15	49
Age of husband	39.664	9.405	17	63
Education of woman				
Uneducated	0.268	0.443	0	1
Primary education	0.411	0.492	0	1
Secondary education	0.295	0.456	0	1
Higher education	0.026	0.16	0	1
Education of partner				
Uneducated	0.175	0.38	0	1
Primary education	0.36	0.48	0	1
Secondary education	0.348	0.477	0	1
Higher education	0.066	0.249	0	1
Occupation status of partner	0.989	0.103	0	1
Religion of woman				
Muslim	0.226	0.418	0	1
Christian	0.708	0.455	0	1
Other	0.066	0.248	0	1
Number of living children	3.467	2.268	0	11
Household wealth				
Poorest	0.207	0.406	0	1
Poor	0.223	0.417	0	1
Average	0.201	0.401	0	1
Rich	0.177	0.382	0	1
Richest	0.192	0.394	0	1
Place of residence: Rural	0.6	0.49	0	1
Exposure to TV	0.555	0.497	0	1
Exposure to Radio	0.495	0.5	0	1
Desire to have no more children	0.335	0.472	0	1
Approval of FP by husband	0.357	0.479	0	1

WBPCI, Woman Bargaining Power Composite Index; FP, family planning.

**Figure 1.**
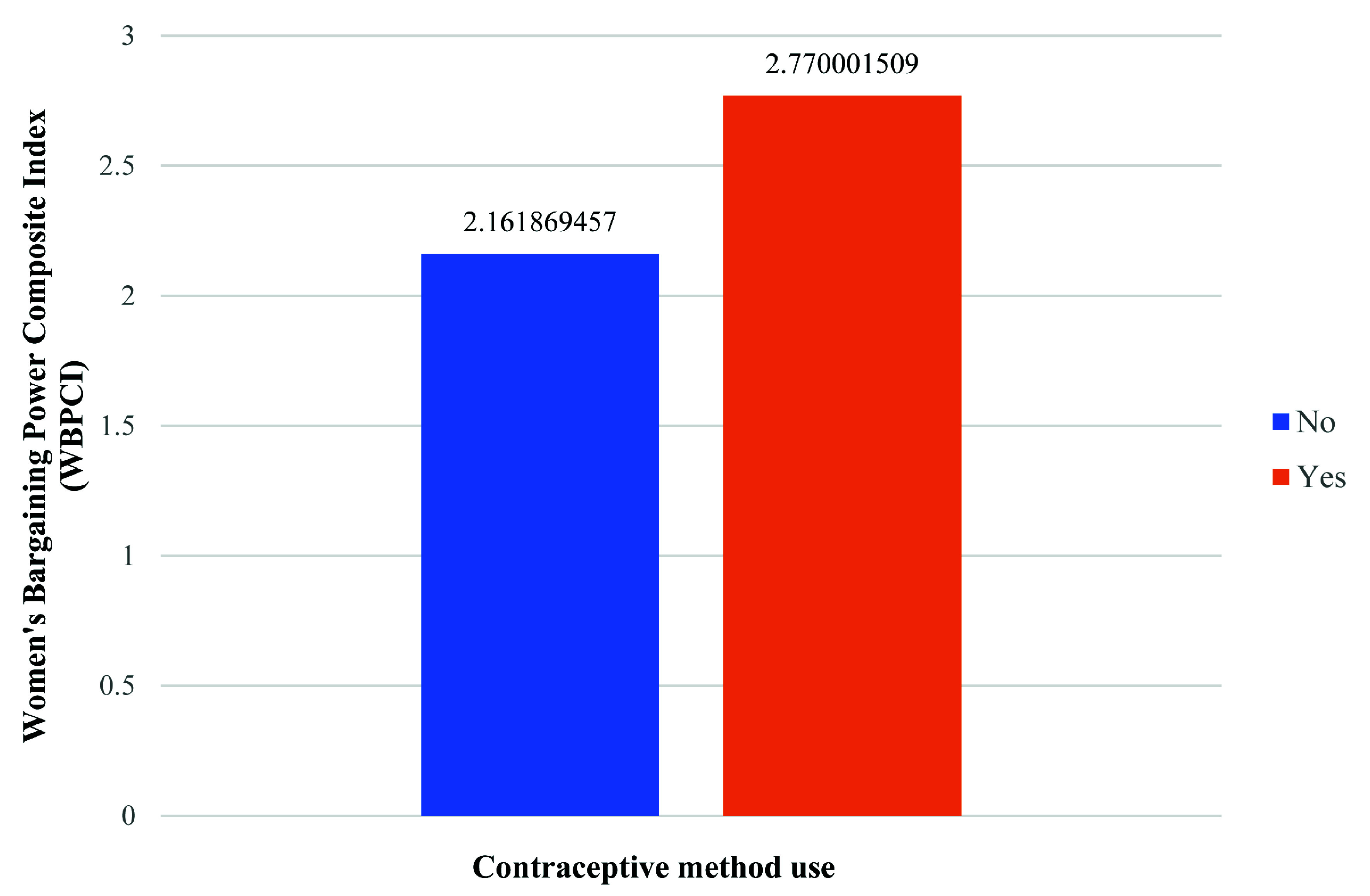
Women’s bargaining power by contraceptive use.

***Bivariate statistics.*** Table 3 breaks down the values and modalities of the different explanatory variables by the use or not of contraception. As expected, focusing on the central variables of the study ( Figure 1), it appears that women's bargaining power is higher among women who use contraception (their average WBPCI is 2.77) than for those who do not use it (their average WBPCI is 2.16). This means that women with high bargaining power are more likely to use contraception than women with low power.

**Table 3.** Cross tabulation between contraceptive use and the explanatory variables

	Contraceptive use		
Variables	Yes		No
		Mean	
*WBPCI*	2.77		2.162
Age of woman	32.254		31.164
Age of partner	40.127		39.526
Number of children	3.795		3.369
		Proportion	
Education of woman			
Uneducated	0.04 ***		0.336
Primary education	0.445 *		0.401
Secondary education	0.466 ***		0.244
Higher education	0.049 ***		0.02
Education of partner			
Uneducated	0.023 ***		0.22
Primary education	0.412		0.344
Secondary education	0.441 ***		0.321
Higher education	0.117 ***		0.051
Occupation status of partner: Employed	0.9895		0.9891
Occupation status of partner: Unemployed	0.0105		0.0109
Religion of woman			
Muslim	0.068 ***		0.273
Christian	0.875 ***		0.658
Other	0.057		0.068
Wealth of household			
Poorest	0.028 ***		0.261
Poor	0.139 ***		0.248
Average	0.214		0.197
Rich	0.268 ***		0.15
Richest	0.351 ***		0.145
Place of residence: Rural	0.442 ***		0.647
Place of residence: Urban	0.558		0.352
Exposure to TV: Yes	0.828 ***		0.474
Exposure to TV: No	0.172		0.525
Exposure to Radio: Yes	0.679 ***		0.441
Exposure to Radio: No	0.321		0.559
Desire to have no more children: Yes	0.453 ***		0.30
Desire to have no more children: No	0.546		0.699
Approval of FP by husband: Yes	0.742 ***		0.243
Approval of FP by husband: No	0.258		0.756

Significance of Chi-square test: *** p<0.01, ** p<0.05, * p<0.1. WBPCI, Woman Bargaining Power Composite Index; FP, family planning.

The average age of women who use contraception is 32 years and 31 years for those who do not use it. Similarly, the spouses of women who use contraception are on average older (40 years) than those of women who do not use contraception (39 years).

Women who used contraception have on average more children than those who do not use contraception (four and three children, respectively). Regarding education, it is noted that among women who use contraception, the proportion of those who have a secondary level of education is the largest (46.6%), followed by primary level (44.45%), higher (4.9%) and those who are uneducated (4%). Conversely, among women who do not use contraception, the proportion of women at the primary level and without education is the highest (40.1% and 33.6%, respectively). The chi-square test reveals a significant relationship (at 1%) between education and contraceptive use. This suggests that as a woman increases further in education, she is more likely to use contraception. Similar findings were made regarding the women’s husbands; the more the husband is educated, the more likely it is that the woman uses contraception. On the other hand, the husband's occupation status is not related to the use of contraception based on the chi-square test result. Among women who use contraception, the proportion of Christians (87.5%) is very high compared to Muslims (6.8%).

With regard to household wealth, the chi-square test leads to a significant relationship at 1% (except average wealth level) with contraception. Thus, the wealthier the household, the more likely the woman uses contraception, because the proportion of women who use contraception is higher among very rich households.

In terms of place of residence, there is also a significant 1% relationship with contraceptive use, and the proportion of women who access contraception is higher in urban areas (55.8%) than in rural areas (44.2%). Women's exposure to the media is significantly associated with contraception. Indeed, among women who use contraception, the proportion of women who are exposed to TV and radio is higher than those who are not exposed (82.8% and 67.9% against 2% and 32.1%, respectively). Finally, the intermediate factors of contraception (the desire to have no more children and the approval of family planning by the husband) are significantly related to the use of contraception.

### Probit model results

Recall that the estimation of the probit model makes it possible to highlight the effect of the woman's bargaining power within the couple on contraceptive use. The results of this estimation are contained in the second column of Table 4. The interpretation of these results can be done from two angles: the effect of the woman's bargaining power and the other determinants of contraceptive use.

**Table 4.** Results of probit model and Heckman probit model.

	Probit	Heckman probit
Variables	dydx	dydx
WBPCI	0.0344 ***	0.0357 ***
	(0.00908)	(0.0105)
Age of woman	0.0119 *	0.0133 *
	(0.00673)	(0.00811)
Age squared	-0.000276 ***	-0.000325 **
	(9.97e-05)	(0.000145)
Education of woman: Primary (Ref=uneducated)	0.0768 ***	0.0851 **
	(0.0290)	(0.0368)
Education of woman: Secondary	0.0829 **	0.0888 **
	(0.0361)	(0.0424)
Education of woman: Higher	0.0411	0.0532
	(0.0626)	(0.0708)
Age of husband	0.000931	0.000820
	(0.00124)	(0.00136)
Education of husband: Primary (Ref=uneducated)	0.133 ***	0.140 ***
	(0.0372)	(0.0419)
Education of husband: Secondary	0.0840 **	0.0896 **
	(0.0375)	(0.0414)
Education of husband: Higher	0.0820	0.0832
	(0.0547)	(0.0582)
Occupation status of husband	0.0410	0.0565
	(0.0452)	(0.0608)
Religion of woman: Muslim	-0.121 ***	-0.137 ***
	(0.0214)	(0.0403)
Religion of woman: Christian	-0.0915 **	-0.102 **
	(0.0357)	(0.0442)
Household wealth: Poor (Ref=poorest)	0.110 ***	0.116 **
	(0.0403)	(0.0461)
Household wealth: Average	0.164 ***	0.171 ***
	(0.0493)	(0.0526)
Household wealth: Rich	0.266 ***	0.277 ***
	(0.0627)	(0.0667)
Household wealth: Richest	0.326 ***	0.332 ***
	(0.0675)	(0.0700)
Place of residence (Ref=Urban)	0.0364 **	0.0392 *
	(0.0183)	(0.0218)
Number of children	0.0209 ***	0.0210 ***
	(0.00443)	(0.00526)
Exposure to TV	0.0650 ***	0.0690 ***
	(0.0196)	(0.0233)
Exposure to radio	0.00684	0.00756
	(0.0150)	(0.0168)
Desire to have no more children	0.0697 ***	0.0881 **
	(0.0210)	(0.0415)
Husband approves FP	0.227 ***	0.244 ***
	(0.0198)	(0.0349)
*N*	2446	2,893
*Prob>chi2*	0.000	0.000
*Pseudo R2*	0.3089	-
*Rho*	-	-0.373
*LR test (Rho=0): Prob>chi2*	-	0.5548

*** p<0.01, ** p<0.05, * p<0.1. dydx, marginal effects; WBPCI, Women’s Bargaining Power Composite Index; FP, family planning; LR, likelihood-ratio.

***The effect of the woman's bargaining power on contraceptive use.*** Table 4 shows that women's bargaining power positively and significantly affects (at 1%) the use of contraception. Indeed, an additional unit of the WBPCI leads to an increase in the probability of using contraception of 0.034. This result is coherent with the results of the bivariate statistics obtained. In the context of Cameroon, this can be explained by the fact that in developing countries, women are allocating more and more time and resources to income-generating activities and education. In fact, the DHS surveys show that the proportion of women at secondary and higher levels who are economically active increased between 2004 and 2011 from 39.1% to 46.2%, respectively. Thus, getting pregnant would be a hindrance to these ambitions. Therefore, if they have high bargaining power, and therefore a strong ability to influence decision-making in the desired direction, the likelihood that they will use contraception will be high. This outcome is similar to that found by Rwenge (2003); Klomegah (2006) and Patrikar *et al.* (2014). On the other hand, it is far from those of DeRose & Ezeh (2010) who found that the decision-making power of the woman (at the individual level) has no effect on the use of contraception in Uganda.

***Other determinants of contraceptive use.*** Age, education, Muslim religion of the woman, household wealth, place of residence, number of living children, exposure to TV, approval of FP by husband and the desire of the woman to have no more children are the other determinants of contraceptive use. The woman's age significantly increases (at 1%) the probability of using contraception by 0.012.

Education of woman is also positively associated with the likelihood of using contraception. The higher the level of education, the higher the positive effect of education on the probability of contraceptive use increases, since the effect of primary education is 0.076 and the effect of secondary education is 0.082. This can be explained by the fact that relative to uneducated women, those who are educated clearly apprehend the importance of using contraception to control her fertility (through spacing or limitation) and to avoid unintended pregnancies. The challenge here is to focus on quality children, that is, children who can attend school and who can receive adequate health care. However, there is the unexpected result that higher education has no significant effect on contraceptive use. With regard to the religion of women, it appears that the Muslim religion negatively and significantly affects the probability of using contraception of 0.121. Indeed, this result can be justified since according to the report of the DHS-MICS 2011, the North Region is not only the area where we find the bulk of the Muslim population (67% in Adamawa Region, 47% in the Far North Region and 39% in the North Region), but it is also the part of the country where the total fertility rate is highest ( average of 5.2 in Adamawa Region, 6.8 in the Far North Region and 6.5 in the North Region). The Christian religion also negatively affects contraception by 0.07.

Household wealth has a positive and significant effect on the probability of contraceptive use, and this positive effect increases as household wealth increases. Compared to households living in urban areas, living in rural areas positively affects the probability of contraceptive use. This may reflect the fact that in rural areas, promotion and counseling campaigns for the use of contraceptive methods are increasingly reinforced and bear fruit. The higher the number of living children of a couple, the more likely the woman will use contraception to reduce the burden she may face. With regard to the exposure variables to the mass media, only exposure to the TV affects contraceptive use, which highlights the strong potential that TV has on an individual’s behaviors with regard to contraception. Finally, all intermediate variables of contraception positively affect the use of contraception. While the desire of a woman to have no more children has an effect of 0.069 on the probability of using contraception, the approval of family planning by husband has a much higher effect (0.239), which stresses the central role that the husband plays as a vector of contraceptive use.

### Robustness checks

The third column of Table 4 also presents the results of the estimation of the probit model with sample selection (Heckman probit model) taking into account the existence of a potential selection bias. The results of the likelihood-ratio test suggest the absence of selection bias since the null hypothesis of a random selection of the sample is accepted (Prob> chi2 = 0.5548). Nevertheless, after controlling for selection bias, the effect of the woman's bargaining power (and other determinants) on the probability of contraceptive use remains virtually unchanged compared to those found in the probit model, which confirms the robustness of previously formulated analyzes. In the literature, Westeneng & D'Exelle (2015) also estimate the Heckman probit model for four equations: contraceptive use, prenatal visits, home delivery and delivery to specialized hospital/center. Regarding the equation of contraceptive use, their analysis also led to the absence of a selection bias.

### Limitations of the study

This work is subject to two main limitations. Firstly, the couple module of the 2011 DHS-MICS data does not include the responses reported by husbands on decision-making in the household. This is why the paper focused on the responses reported by women only. This approach may be biased to the extent that these responses are likely to mask the reality of the couple's life and the true status of each spouse within the household. Secondly, women's bargaining power may be related to certain unobservable factors that also influence contraceptive use, suggesting the potential endogeneity of bargaining power. This aspect has been neglected in the analysis produced.

## Conclusions

The objective of this paper was to assess the effect of women's bargaining power on contraceptive use based on Demographic and Health Survey and Multiple Indicators realized in 2011 in Cameroon. The bargaining power of women is measured by the WBPCI, constructed by a multiple correspondence analysis on the indicators that make up the six dimensions selected for its apprehension: the role in the decision-making on key aspects of household life, control over financial resources, economic position in the household, women's attitudes towards husband's violence, membership to associations and the interruption of the interview by an adult. Given the binary nature of contraceptive use, the probit model was used as the econometric method. The results obtained from bivariate statistics suggest that women's bargaining power is higher for women who use contraception than for women who do not use contraception. As for the econometric results, especially those of the probit model estimation, it appears that the increase of the woman's bargaining power in the couple significantly increases the probability of contraceptive use. In addition, education (primary and secondary), household wealth, exposure to TV media and intermediate variables of contraception are other determinants of contraceptive use.

Elsewhere, a robustness analysis was performed. To measure the effect of women's bargaining power on contraceptive use, women who were pregnant at the time of the survey had to be excluded, which suggests potential selection bias. Thus, a probit model with sample selection (Heckman probit model) was estimated to take into account the plausible selection bias of the sample. The outcome of the likelihood ratio test revealed the absence of selection bias. The results obtained from this Heckman probit model converge on the effect of women's bargaining power on contraceptive use, which is almost unchanged compared to that obtained from the probit model, which makes the conclusions of the probit robust.

Finally, this research is relevant in terms of economic policy implication. Indeed, the public health policy in force aims to significantly reduce the high maternal mortality rates by significantly increasing the number of women on contraception from 16.1% in 2011 to 30% by 2020. Firstly, since our findings postulate that women with high bargaining power are able to use the most appropriate reproductive health care, policy makers need to look at the social policies of status revitalization and women's empowerment within households with much acuity. Secondly, policy makers need to offer more opportunities and educational resources to encourage women to progress in education.

## Data availability

### Underlying data

The 2011 Cameroon Demographic and Health and Multiple Indicators Survey dataset used for this study is available online from the DHS website under the *Couples’ Recode* subsection:

https://dhsprogram.com/data/dataset/Cameroon_Standard-DHS_2011.cfm?flag=1. Data can be accessed by applying through the DHS website. Registration is required and access is granted for legitimate research purposes. Further information about data access can be found at: https://dhsprogram.com/data/Access-Instructions.cfm.

### Extended data

Figshare: Extended data of the paper "The effect of women's bargaining power within couples on contraceptive use in Cameroon". https://doi.org/10.6084/m9.figshare.11719497 ( Tchakounte, 2020)

Data are available under the terms of the Creative Commons Zero "No rights reserved" data waiver (CC0 1.0 Public domain dedication).

## Software availability

Source code available from: https://github.com/DimitriTchakounte/Stata-Do-file.git

Archived source code at time of publication: https://doi.org/10.6084/m9.figshare.11371680.v2 ( Tchakounte, 2019)

License: MIT

## Footnotes

1 Bargaining power is defined as the ability of an individual to influence household decision-making. It is the amount of influence an individual has over other individuals in the household ( Doss, 2013).

2 The current maternal health improvement policy is contained in the 2015–2020 National Family Planning Operational Plan with the primary goal of increasing the number of women on contraception from 16% in 2011 to 30% by 2020.

3 The six dimensions selected have a total of 31 indicators. After applying the principle of First Axis Ordering Consistency (FAOC) as prescribed by Asselin (2002), from the results of the first iteration of the MCA, it appears that three of these indicators (those of the interruption of the interview) are not appropriate for the construction of the WBPCI. They were thus subtracted from the second and last iteration.

4 The wealth index is generated by the DHS program using principal component analysis using data on a household’s ownership of selected assets, such as televisions and bicycles; materials used for housing construction; and types of water access and sanitation facilities.

5 According to the classification of the National Institute of Statistics, the urban area includes cities of at least 10,000 inhabitants and, the rural area includes small agglomerations of less than 10,000 inhabitants.
