# The effect of women’s bargaining power within couples on contraceptive use in Cameroon

**DOI:** 10.12688/gatesopenres.13100.3

**Published:** 2024-07-29

**Authors:** Dimitri Tchakounté Tchuimi, Benjamin Fomba Kamga

**Affiliations:** 1 Faculty of Economics and Management, University of Yaounde II, Yaounde, Centre, Po. Box 1792, Cameroon

**Keywords:** Women’s bargaining power, Contraceptive use, Couple, Maternal death, Probit model, Cameroon

## Abstract

**Background:**

The prevalence of contraception among married women, evaluated at 23%, is low in Cameroon. Maternal death rates, estimated at 782 deaths per 100,000 live births, are very worrying. The National Strategic Plan for Reproductive, Maternal, Newborn and Child Health (2015–2020) and the Health Sector Strategy (2016–2027) focuses on increasing modern contraceptive prevalence as a means to reduce maternal death. This paper identifies women’s bargaining power as a factor that may stimulate contraceptive use. The objective of this study is to analyze the association between women's bargaining power within couples and modern contraceptive use.

**Methods:**

The data used come from the fifth Demographic and Health Survey (DHS) conducted in 2018. Women’s bargaining power within couple is measured by a Woman Bargaining Power Composite Index (WBPCI) built through a multiple correspondence analysis. The logistic regression model was used to analyze the relationship between WBPCI and modern contraceptive use.

**Results:**

The results of the descriptive statistics show that women's bargaining power is higher among women who use contraception than for those who do not. The results of the logistic regression model show that an increase of WBPCI was significantly associated with higher chances of using a modern contraceptive method (OR = 1.352; 95% CI: 1.257, 1.454; p <0.01). The education of women is also a key determinant since educated women were at least two times more likely to use a modern contraceptive method than uneducated women.

**Conclusions:**

To reduce high maternal death rates in Cameroon, public health policies should not only focus on the health system itself, but should also focus on social policies to empower women in the household.

## Introduction

Improving maternal health is one of the key priorities of public health policies in the world in general, especially in developing countries. Defined in 2000 as the fifth Millennium Development Goal (MDG-5), the reduction of maternal deaths was renewed in 2015 as a target of the third Sustainable Development Goal (SDG-3). For a woman, pregnancy is a period of the life cycle during which her health is particularly vulnerable. In fact, the complications observed throughout this period seem to be the main cause of maternal deaths in developing countries ( Haider *et al.*, 2017). The use of reproductive health care services, particularly in relation to the use of contraception to control fertility and, to prevent early and unwanted pregnancies, appears to be an investment to ensure good health for the mother. Thus, access to reproductive health care is an effective weapon for limiting the risk of maternal morbidity and death ( Hou & Ma, 2013).

In developing countries, several factors may be associated with the use of reproductive health care services. The 1994 International Conference on Population and Development (ICPD) in Cairo already emphasized the role of women's economic empowerment. Moreover, as it is recognized that decisions that lead women to use reproductive health care services occur in the sphere of marriage, the household or the family ( Becker, 1996), much literature has suggested that appreciation of women's bargaining power ^1^, while at the same time reducing gender inequalities in the couple, is an important driver for the use of reproductive health care service ( Blanc, 2001; Chapagain, 2006; Haque *et al.*, 2012; Kamiya, 2011). It is generally recognized that women's bargaining power over resource allocation and decision-making is assessed through so-called modern factors, such as education, income, possession of property, legislation prohibiting violence against women and the freedom to move about ( Adjiwanou *et al.*, 2018; Allendorf, 2007; Beegle *et al.*, 2001; Habibov *et al.*, 2017). However, the power of women is hardly indissociable from the channel of social and cultural norms on the allocation of resources in the household and the role of women in the organization of the household and the couple; in most cases, these standards restrict women in decision-making both for themselves (decisions about the use of adequate health care, among others) and their children. As a result, since women's traditional values may hinder the positive impact of their modern values, the mechanisms by which women's bargaining power can influence the use of reproductive care appear delicate and ambiguous ( Westeneng & D'Exelle, 2015), drawing our attention to the specificity of the context in which the study is conducted.

In Cameroon, the assessment of the use of reproductive health care is of particular importance since maternal death levels, estimated at 467 deaths per 100,000 births by the Demographic and health Survey (2018), are very worrying. Current government policy on reproductive health focuses on facilitating access to reproductive care (with a focus on assessing modern contraceptive prevalence) as a means of reducing these high rates of maternal mortality. However, it is becoming increasingly clear that issues related to reproductive health are more of a joint behavior in a couple than an individual choice. Indeed, in Africa, marriage remains the privileged place of procreation ( Noumbissi & Sanderson, 1999) and where the decision to use adequate reproductive health care results from the ability of each partner expressing preferences through negotiation ( Nikiema *et al.*, 2008).

This paper aims to analyze the association between women's bargaining power within couples and contraceptive use in Cameroon. The contribution of this study is at two main levels. First, information from work linking women's bargaining power in the couple and reproductive health is fairly abundant in countries with a strong tendency to conserve patriarchal cultures. However, this type of investigation is particularly rare in Cameroon, a country not bordering on the prevalence of asymmetrical gender relations and power imbalances between spouses. This study will fill the gap related to this information scarcity. Secondly, this study aims to provide an effective tool for achieving the goal of reducing maternal death rates of the current health policy in the horizon of 2027 ^2^.

## Methods

### Ethical statement

Permission to use the datasets for this research was obtained prior to the study from the Demographic and Health Surveys (DHS) Program. Ethical approval was not required for this study because it used anonymized DHS data only. The IRB-approved procedures for DHS public-use datasets do not in any way allow respondents, households, or sample communities to be identified.

### Data

The data came from the fifth DHS carried out in 2018 by the National Institute of Statistics (INS) in collaboration with the Ministry of Public Health and financial support from the United States Agency for International Development (USAID), United Nations Population Fund (UNFPA) and UNICEF. As in other developing countries, this survey has collected information on household characteristics, reproductive and sexual health/AIDS, children's health, STD/AIDS knowledge, attitudes and behaviors, and the bargaining power of women in households. Three questionnaires were used to collect the data in this survey: the women's questionnaire, the men's questionnaire and the household questionnaire. These different questionnaires generated five modules: the child, woman, man, household and couple modules. We use woman module within which the answers to the questions were reported by women aged 15 to 49.

In total, a representative sample of 14,677 women were constituted at the end of the survey. However, two restriction criteria must be specified. Firstly, the study considers only married women or women living in a union with a partner since participation in decision-making, which is an important dimension of women's bargaining power, only concerns women in a couple. Thus, 6,617 women (comprised of 4,915 never in union, 757 widowed, 237 divorced and 708 separated) are excluded from the sample. Secondly, since we are interested in the use of contraception at the time of the DHS survey, the analysis is limited to couples in which women were not pregnant. The data comprised of a total of 1,514 women where pregnant at that time was therefore excluded. Thus, the analysis rely on a final sample consists of 6,546 married women.

### Variable measurement

***Dependent variable.*** The dependent variable of this paper is contraceptive use. It is measured by a variable that takes the value 1 if the woman uses a modern contraceptive method at the time of the survey, and 0 if not (traditional method or no method). The modern contraceptive methods include the pill, the intra-uterine device (IUD), the injection, the male condom, the implant/Norplant, the female condom, the female sterilization, the male sterilization, emergency contraception, standard days method and other modern method. The traditional methods include periodic abstinence, withdrawal, breastfeeding, and lactational amenorrhea (LAM).

***Independent variables***

The variable of interest, women’s bargaining power:

The transition from the theoretical conception of the woman's bargaining power to her empirical measure is associated with many difficulties since the process of negotiation between the partners within a couple is unobservable ( Beegle *et al.*, 2001; Friedberg & Webb, 2006). Thus, to measure the bargaining power of women, it is essential to resort to proxies ( Doss, 2006). A review of the literature reveals that several dimensions can be used to design it ( Ewerling *et al.*, 2017; Hanmer & Klugman, 2016; Lépine & Strobl, 2013; Mahmud *et al.*, 2012).

In this research, women's bargaining power is apprehended through four specific dimensions: *(i)* economic status/independence in the household, *(ii)* control over financial resources, *(iii)* decision-making on key aspects of household life, and *(iv)* women's attitudes towards husband’s violence (a dimension to understand the contribution of cultural norms to the role of gender justifying the domination of the husband over the women). The dimension of the woman's economic status is made up of three indicators: whether the mother worked during the 12 months preceding the survey; whether the mother owned a house, and whether the mother owned land. The dimension of control over financial resources has four indicators: The person who decides how to spend mother's income; the person who decides how to spend husband's income; whether the mother used telephone for financial transactions; whether the mother had an account in a bank or other financial institution. The dimension of decision-making contains four indicators: the person who decides on the mother's health care; the person who decides on contraceptive use; the person who decides on general household purchases and the person who decides on visits to family and relatives. The last dimension, women's attitudes towards husband’s violence, contains five indicators: wife-beating is justified if wife goes out without telling husband; wife-beating is justified if wife neglects children; wife-beating is justified if wife argues with husband; wife-beating justified if wife refuses to have sex with husband; wife-beating justified if wife burns the food.

Table A1 ( Tchakounte, 2021b) in the appendix provides a detailed description of each of these dimensions and the codes of indicators. A multiple correspondence analysis (MCA) on the indicators that makes up each of these dimensions has permitted us to generate a Women Bargaining Power Composite Index (WBPCI) used in our investigations to measure the bargaining power of women. The MCA is a factorial method allowing to synthesize the common information shared by several dimensions or several indicators ( Greco *et al.*, 2019). The choice of this method is based on the idea according to which the indicators selected cannot be considered as equivalent in the information they provide on women’s bargaining power. It would then be relevant to reflect this idea in the structure of the constructed index. Practically, the WBPCI is constructed through the first factorial axis from the MCA. The results of this MCA are contained in the Table A2 ( Tchakounte, 2021b) of the Appendix ^3^. It comes out that the total inertia explained by the factorial plan is 82,21%, that is 61.49% explained by the first factorial axis and 20.71% explained by the second factorial axis.

Keeping the continuous values of the WBPCI has the relevance of avoiding ambiguities related to a categorization of this index in various modalities ( Haider *et al.*, 2017). As Richardson (2018) points out, using threshold values to categorize such an index is not appropriate as it is only a matter of value judgments and a subjective approach that sometimes may not reflect the context in which the study is conducted.

In addition, Cronbach's alpha coefficient is used to measure the internal consistency of the WBPCI. The closer the value of this coefficient is to 1, the more reliable the constructed index is ( Aiken & West, 1991). Here, the value of Cronbach's alpha obtained is 0.7262, which indicates the reliability of the WBPCI since the value of alpha is upper than 0.70 ( Nunnally, 1978).

Control variables:

Control variables are divided into three categories: socio-demographic, economic and cultural factors, media exposure factors, and intermediate variables of contraception. The first category of factors includes age, education, work status, religion, number of living children, household wealth and place of residence. Three factors of women’s exposure to the media are considered: exposure to television, exposure to radio and exposure to newspapers. For the third category of factors, only one intermediate variable of contraception is considered: the desire of the woman not to have an additional child. Table 1 below highlights the description, measurement and codes of control variables.

**Table 1.** Description, measurement and codes of control variables.

Variables	Description and measurement
***Sociodemographic, economic and cultural factors***	
Age of woman	Age (number of years) of the woman at the time of the survey measured by three dummy variables: 15–24, 25–34, and 35–49.
Education of woman	The highest level of education attained by women measured by four dummy variables: uneducated, primary, secondary and higher level.
Education of partner	The highest level of education attained by partner measured by four dummy variables: uneducated, primary, secondary and higher level.
Status of occupation of husband	Measured by a binary variable taking the value 1 if the husband worked in the 12 months preceding the survey and 0 if not.
Religion of woman	Represents the religious affiliation of the woman measured by three dummy variables: Muslim, Christian and other religions.
Number of living children	Represents the number of living children of the couple, and measured by 5 dummy variables: 0, 1-2, 3-4, 5-6 and more than 6.
Household wealth	Represents the household's living standard and measured the wealth index, which categorizes household into five wealth quintiles: poorest, poor, average, rich and richest ^4^.
Place of residence of household	Measured by a binary variable taking the value 1 if the household lived in a rural area and 0 if they lived in an urban area ^5^.
***Factors of exposure to media***	
Exposure to TV	Number of times the woman watched TV per week and measured by a dummy variable: 1 if at least once per week and 0 if not once.
Exposure to radio	Number of times the woman listened to the radio per week and measured by a dummy variable: 1 if at least once per week and 0 if not once.
Exposure to newpapers	Number of times the woman read newspapers per week and measured by a dummy variable: 1 if at least once per week and 0 if not once.
***Intermediate variables of contraception***	
Desire of the woman to have no more children	Measured by a dummy variable taking the value 1 if the woman no longer wanted to have children and 0 if not.

### Empirical methods

In addition to descriptive statistics, this paper also uses an econometric model. To this end, it is worth recalling that the objective of the research is to analyze the association of women's bargaining power within couples with contraceptive use. Since contraceptive use is a dichotomous variable, a qualitative dependent variable model, and more specifically a logistic regression model, is used.

***Specification of logistic regression model.*** The logistic regression model can be formalized using the following equation:

$$\log\left(p/1-p\right)=\beta_{0}+\beta_{1}WBP_{i}+\beta_{2}X_{i}+\varepsilon_{i}\;(1)$$

Where ( *p*/1– *p*) represents the chances of using a modern contraceptive method by the woman *i*. *WBP _i_* corresponds to the bargaining power of the woman *i* and is measured by WBPCI. *β* is the coefficient which allows measurement of the association between the woman’s bargaining power and modern contraceptive use. *X* is the matrix of control variables, and *γ* is the vector of parameters which permits assessment of the relationships between the control variables contained into *X* and contraceptive use. *Ԑ* is the error term independently and identically distributed. The regression results were presented by the estimated odds ratio (OR) and adjusted odds ratio (AOR) with 95% confidence interval (95% CI).

***Software.*** The software used to carry out the empirical analyzes of this study is STATA, version 15 ( StataCorp, 2017).

## Results

### Results of descriptive statistics

Table 2 below highlights the descriptive statistics of the variables of the study. It appears that among married women at the time of the survey, only 17.7% used a modern contraceptive method, 5.68% used a traditional method and 76.58% were not using any method.

**Table 2.** Descriptive statistic of variables.

Variables	Mean/proportion	Std. Dev.	Min	Max
Contraceptive use (modern)	0.177	0.42	0	1
WBPCI	0.134	0.943	-2.79	1.586
Age of woman				
15–19	0.071	0.258	0	1
20–24	0.152	0.359	0	1
25–29	0.207	0.405	0	1
30–34	0.194	0.395	0	1
35–39	0.158	0.365	0	1
40–44	0.12	0.325	0	1
45–49	0.097	0.296	0	1
Education of woman				
Uneducated	0.251	0.434	0	1
Primary education	0.328	0.469	0	1
Secondary education	0.369	0.482	0	1
Higher education	0.052	0.223	0	1
Education of partner				
Uneducated	0.231	0.422	0	1
Primary education	0.312	0.463	0	1
Secondary education	0.369	0.483	0	1
Higher education	0.088	0.283	0	1
Occupation status of partner	0.968	0.177	0	1
Religion of woman				
Muslim	0.279	0.449	0	1
Christian	0.684	0.465	0	1
Other	0.037	0.189	0	1
Number of living children				
0	0.068	0.252	0	1
1–2	0.33	0.47	0	1
3–4	0.311	0.463	0	1
5–6	0.193	0.395	0	1
6+	0.099	0.298	0	1
Household wealth				
Poorest	0.174	0.379	0	1
Poor	0.21	0.407	0	1
Average	0.233	0.423	0	1
Rich	0.201	0.401	0	1
Richest	0.182	0.386	0	1
Place of residence: Rural	0.523	0.499	0	1
Exposure to television	0.514	0.5	0	1
Exposure to radio	0.377	0.485	0	1
Exposure to newspaper	0.163	0.37	0	1
Desire to have no more children	0.338	0.473	0	1

WBPCI, Woman Bargaining Power Composite Index.

Concerning the bargaining power of women within the couple, the average value of WBPCI is 0.134 (knowing that the maximum value is 1.586 and the minimum is -2.79). Looking at control variables, 20.7% of married women were 25–29 of age, 19.4% were aged 30–34, 15.8% were aged 35–39, 15.2% were aged 20–24, 12% were aged 40–44, 9.7% were aged 45–49, and only 7.1% were 15–19 of age. Among these women, the most representative level of education is the secondary level (36.9%), followed by the primary level (32.8%), uneducated (25.1%) and the higher level (5.2%). In addition, most of partners have a secondary level of education (36.9%), followed by primary level (31.2%), uneducated (23.1%) and the higher level (8.8%). Almost all partners (96.8%) had an occupation in the 12 months preceding the survey. The most prevalent religion among married women is Christianity (68.4%), with 27.9% of Muslim religion. 33% of women have 1–2 living children with their partners, 32.1% have 3–4 living children, 19.3% have 5–6 living children, 9.9% have more than 6 children and 6.8% have no living children. Regarding household wealth, 23.3% of average wealth, 21% of household were poor, 20.1% were rich, 18.2% were very rich and 17.4% were very poor. In addition, 52.3% of households lived in rural areas. Regarding media exposure variables, 51.4% of married women watched TV at least once a week, 37.7% listened to the radio at least once a week and 16.3% read newspapers at least once a week. Finally, for the intermediate variables of contraception, 33.8% of married women no longer wanted to have children.

### Logistic regression model results

Recall that the estimation of the logistic regression model makes it possible to highlight the association of the woman's bargaining power within the couple with contraceptive use. The results of this estimation are contained in the Table 3. This table have two models: an unadjusted model and an adjusted model. The unadjusted model is the one that includes only women’s bargaining power as the independent variable, while the adjusted model includes control variables in addition to bargaining power of women. The results of the unadjusted model estimation illustrate that women’s bargaining power was significantly associated with the likelihood of using a modern contraceptive method. Specifically, it emerges that an increase in the WBPCI was associated with higher chances of contraceptive use (OR = 1.352; 95% CI: 1.257, 1.454; p <0.01). From the adjusted model, the association between WBPCI and contraceptive use is not more significant. By looking at other determinants of contraceptive use, it emerges that the woman’s age and religion, husband’s occupation and the place of residence (rural) were significantly associated with lower odds of using a modern contraceptive method, while women’s education, husband's education (higher), household wealth (poor), number of children (3–4, 5–6, 6+), and exposure to radio and newspapers were significantly associated with higher odds of using modern contraception.

**Table 3.** Results of logistic regressions on contraceptive use.

	Unadjusted Model		Adjusted Model	
VARIABLES	OR	95%CI	OR	95%CI
*WBPCI*	1.352 ***	(1.257, 1.454)	0.996	(0. 918, 1.081)
Age of woman (ref=15–24)				
25–34			0.602 ***	(0.503, 0.719)
35–49			0.260 ***	(0.208, 0.323)
Education of woman (ref=uneducated)				
Primary			2.307 ***	(1.7955, 2.962)
Secondary			2.802 ***	(2.113, 3.712)
Higher			2.685 ***	(1.775, 4.059)
Education of husband (ref=uneducated)				
Primary			1.188	(0.949, 1.485)
Secondary			1.116	(0.884, 1.409)
Higher			1.375 *	(0.993, 1.902)
Occupation status of husband			0.391 ***	(0.308, 0.495)
Religion (ref=other)				
Muslim			0.149 ***	(0.111, 0.198)
Christian			0.323 ***	(0.246, 0.422)
Household wealth (ref=poorest)				
Poor			1.262 *	(0.982, 1.620)
Middle			1.197	(0.912, 1.571)
Richer			1.059	(0.768, 1.460)
Richest			1.061	(0.739, 0.1521)
Place of residence (ref=urban)			0.531 ***	(0.445, 0.633)
Number of children (ref=none)				
1–2			0.948	(0.767, 1.169)
3–4			1.570 ***	(1.256, 1.962)
5–6			1.987 ***	(1.517, 2.601)
6+			2.098 ***	(1.482, 2.968)
Exposure to TV			0.961	(0.781, 1.181)
Exposure to Radio			1.302 ***	(1.112, 1.522)
Exposure to News Paper			1.301 ***	(1.083, 1.562)
Desire to have no more children			1.380 ***	(1.159, 1.641)
Observations	6,546		6,546	
Prob > chi2	0.000		0.000	
Log pseudolikelihood	-3025.17		-2797.14	
Model AIC	6054.35		5644.28	

*** p<0.01, ** p<0.05, * p<0.1. dydx, marginal effects. WBPCI, Woman Bargaining Power Composite Index.

## Discussion

### The association of the woman's bargaining power with contraceptive use

The results obtained from the logistic regression model (first column of the Table 3) illustrate that women's bargaining power within couples was significantly associated with higher chances of using a modern contraceptive method. This result can be explained by the fact that in developing countries, women are allocating more and more time and resources to income-generating activities and education. In fact, the Cameroon DHS surveys show that the proportion of women at secondary and higher levels who are economically active increased between 2004 and 2018 from 39.1% to 51.3% respectively (and 46.2% in 2011). Thus, getting pregnant would be a hindrance to these ambitions. Therefore, if they have high bargaining power, and therefore a strong ability to make choices and influence decision-making in the desired direction, the likelihood that they will use contraception will be high. This result is similar to that found by Klomegah (2006); Patrikar *et al.* (2014); Rwenge (2003), and recently by Juraqulova & Henry (2020) who found that in Tajikistan the probability of using contraception is higher for a woman who has a say in the decision to control births and has both control over her own health care and financial means to get medical help than a woman who does not have these choices. On the other hand, it is far from those of DeRose & Ezeh (2010) who found that the decision-making power of the woman (at the individual level) has no effect on the use of contraception in Uganda. However, when controlling for covariates, the association between the bargaining power of the woman with contraceptive use lose its significance.

### Other determinants of contraceptive use

Compared to women aged 15–24, women aged 35–49 and 25–34 have lower chances to use modern contraception, suggesting that the older the woman, the more her capacities of procreation decrease, and these capacities are canceled out when she is menopaused. Education of woman is also a key determinant of contraceptive use. In fact, compared to uneducated women, women with primary, secondary or higher education have higher chances to use modern contraception. This can be explained by the fact that relative to uneducated women, those who are educated clearly apprehends the importance of using contraception to control her fertility (through spacing or limitation) and to avoid unintended pregnancies. The challenge here is to focus on quality children, that is, children who can attend school and who can receive adequate health care. With regard to the religion of women, it appears that the Muslim religion was significantly associated with lower odds of contraceptive use. Indeed, this result can be justified since according to the report of the DHS 2018, the North Region is not only the area where we find the bulk of the Muslim population (75.13% in Adamawa region, 52.33% in the Far-North region and 48.63% in the North region), but it is also the part of the country where the total fertility rate is highest (5.9 on average in the Far-North, 6.2 in the North and 4.6 in Adamaoua). The Christian religion was also associated with low chances of modern contraception.

Women have greater chances to use modern contraception when their husbands are educated, compared to women with uneducated husbands. But the husband’s occupation status was significantly associated with lower odds of contraceptive use. Relatively to household wealth, women from poor households have higher chances of contraceptive use compared to women from poorest households. Moreover, the likelihood of using modern contraception is low in households living in rural areas, compared to those living in urban areas. This may reflect the fact that the impact of promotion and counseling campaigns for the use of contraceptive methods are weak in rural areas. The higher the number of living children of a couple, the more likely the woman will use contraception to reduce the burden she may face. With regard to the exposure variables to the mass media, exposure to radio and exposure to newspapers are associated with higher odds of contraceptive use, which highlights the strong potential that these medias have on an individual’s behaviors with regard to contraception. Finally, the desire of a woman to have no more children was linked to lower probability of using contraception.

### Limitations of the study

This work is subject to two main limitations. Firstly, the *woman* module of the 2018 DHS data does not include the responses reported by husbands on decision-making in the household. This is why the paper focused on the responses reported by women only. This approach may be biased to the extent that these responses are likely to mask the reality of the couple's life and the true status of each spouse within the household. Secondly, women's bargaining power may be related to certain unobservable factors that also influence contraceptive use, suggesting the potential endogeneity of bargaining power. This aspect has been neglected in the analysis produced. Despite these limitations, this is the first study to analyze the relationship between women’s bargaining power and modern contraception in Cameroon, using a recent and credible dataset. It therefore constitutes a reference for several future investigations.

## Conclusions

The objective of this paper was to analyze the association of women's bargaining power within couples with contraceptive use based on Demographic and Health Survey realized in 2018 in Cameroon. Contraceptive use is measured by a binary variable (modern contraception or not), and the bargaining power of women is measured by WBPCI, constructed by a multiple correspondence analysis on the indicators that make up the four dimensions selected for its apprehension: the role in the decision-making on key aspects of household life, control over financial resources, economic position in the household and women's attitudes towards husband's violence. The logistic regression model was used as the econometric method, and the results suggest that, without covariates, the woman's bargaining power within the couple was significantly associated with higher chances of using a modern contraceptive method. However, when the control variables were introduced in the model, it emerges that women's bargaining power no longer has a significant influence on contraceptive use. The mother's level of education was found to be the factor with the greatest significant influence on the likelihood of using contraception. The religion of women, husband’s education (higher) and occupation status, household wealth, place of residence, exposure to medias (radio, and newspapers) the desire of a woman to have no more children of contraception are other determinants of contraceptive use.

Finally, this research is relevant in terms of economic policy implication. Indeed, the public health policy in force aims to significantly reduce the high maternal mortality rates by significantly increasing the number of women on modern contraception from 16.1% in 2011 to 35% by 2027. Firstly, since our findings postulate that women with high bargaining power are able to use the most appropriate reproductive health care, policy makers need to look at the social policies of status revitalization and women's empowerment within households with much acuity. Secondly, policy makers need to offer more opportunities and educational resources to encourage women to progress in education.

## Data Availability

The 2018 Cameroon Demographic and Health and Multiple Indicators Survey dataset used for this study is available online from the DHS website under the *Woman’s Recode* subsection: https://dhsprogram.com/data/dataset/Cameroon_Standard-DHS_2018.cfm?flag=1. Data can be accessed by applying through the DHS website. Registration is required and access is granted for legitimate research purposes. Further information about data access can be found at : https://dhsprogram.com/data/Access-Instructions.cfm. Figshare: Extended data of the paper "The effect of women's bargaining power within couples on contraceptive use in Cameroon". https://doi.org/10.6084/m9.figshare.11719497 ( Tchakounte, 2021b). Data are available under the terms of the Creative Commons Zero "No rights reserved" data waiver (CC0 1.0 Public domain dedication).

## Footnotes

^1^ Bargaining power is defined as the ability of an individual to influence household decision-making. It is the amount of influence an individual has over other individuals in the household ( Doss, 2013).

^2^ The current maternal health improvement policy is contained in the Health Sector Strategy 2016-2027 with the primary goal of increasing the number of women using modern contraception from 16% in 2011 to 35% by 2027.

^3^ The four dimensions selected have a total of 16 indicators. After applying the principle of First Axis Ordering Consistency (FAOC) as prescribed by Asselin (2002), from the results of the first iteration of the MCA, it appears that all these indicators are appropriate for the construction of the WBPCI.

^4^ The wealth index is generated by the DHS program with a principal component analysis using data on a household’s ownership of selected assets, such as televisions and bicycles; materials used for housing construction; and types of water access and sanitation facilities.

^5^ According to the classification of the National Institute of Statistics, the urban area includes cities of at least 10,000 inhabitants and, the rural area includes small agglomerations of less than 10,000 inhabitants.

## Software availability

Source code available from: https://github.com/DimitriTchakounte/Stata-Do-file.git

Archived source code at time of publication: https://doi.org/10.6084/m9.figshare.11371680 ( Tchakounte, 2021a)

License: MIT License
