# Supplementary material for: The effect of women’s bargaining power within couples on contraceptive use in Cameroon
Source: Gates Open Res. 2024 Jul 29;4:20. Originally published 2020 Feb 10. [Version 3] doi: 10.12688/gatesopenres.13100.3 (PMC11259594; doi:10.12688/gatesopenres.13100.3)
Supplement: Peer review history

# Reviewer report: Reviewer response for version 3

**DOI:** 10.21956/gatesopenres.17621.r37583

**Published:** 2024-08-06

**Authors:** Manini Ojha

**Affiliations:** 1 O. P. Jindal Global University, Sonipat, Haryana, India

Most of my previous comments have not been addressed by the authors in the version I have received. I cannot approve the manuscript for indexing with those comments and suggestions unaddressed.

Is the work clearly and accurately presented and does it cite the current literature?

Yes

If applicable, is the statistical analysis and its interpretation appropriate?

Partly

Are all the source data underlying the results available to ensure full reproducibility?

Yes

Is the study design appropriate and is the work technically sound?

Yes

Are the conclusions drawn adequately supported by the results?

Partly

Are sufficient details of methods and analysis provided to allow replication by others?

No

Reviewer Expertise:

Applied microeconometrics, empirical development economics, economics of the households, health, gender and education

I confirm that I have read this submission and believe that I have an appropriate level of expertise to state that I do not consider it to be of an acceptable scientific standard, for reasons outlined above.

# Reviewer report: Reviewer response for version 2

**DOI:** 10.21956/gatesopenres.14509.r36171

**Published:** 2024-07-23

**Authors:** Manini Ojha

**Affiliations:** 1 O. P. Jindal Global University, Sonipat, Haryana, India

**Referee Report**

**The effect of women’s bargaining power within couples on contraceptive use in Cameroon**

 The paper uses DHS data to address whether women’s bargaining power within couples plays a role in modern contraceptive use in Cameroon. This is an important question worthy of research, especially since there is a gap in the literature as far as Cameroon is concerned.

 I have now read the paper with great interest and in detail. However, I have certain reservations about the paper and I provide detailed comments on what can be done to improve the paper. I recommend a major revision and resubmission for the paper.

  

1. Page 3, “Moreover, as it is recognized……..health care service (Blanc, 2001…..)” This sentence is long winding and awkward. This needs to be rephrased.
2. Data: the study utilizes only married women or women living in a union with a partner. I’m curious to know whether these women are married only once or more than once. It would be good to see the results for the sample of women married only once to ensure that responses are for the current union only.
3. The authors’ outcome variable is contraceptive use defined as a binary taking value 1 if she uses modern contraceptive and 0 if she uses traditional or no contraception at all. However, traditional forms of contraception are also methods of contraception. I would be interested in seeing the analysis if the author code the contraceptive use variable as 1 for modern contraceptive vs 0 for no contraceptives; 1 for modern contraceptive & 0 for traditional contraceptives; 1 for modern & traditional contraceptives & 0 for no contraceptives. I advise the authors to add this analysis for sensitivity checks.
4. It would be useful for the authors to explain in simple words how to interpret the WBPCI that is constructed through MCA.
5. Why does the analysis not include any information on the woman’s husband characteristics? I believe as robustness, additional controls including the husband’s characteristics – atleast his employment, age, education ought to be included. The “descriptive statistics” show some summaries for partner characteristics but it is not clear if these are included in the regressions from the section on “Control variables”. In addition, DHS has information on whether the husband desires additional kids or not. This ought to be included in the regression as well as use of contraception may be a joint decision as explained by the authors.   
6. I believe there is some information available in the DHS about women’s health care utilization. It would be interesting to see the results upon inclusion of some of these variables – doctor’s visit, distance to health facility and so on. Such variables also belong to the model that determines use of contraception.
7. On Page 5, under the “specification of logistic regression model”, there is a typo “?” while describing the error term.
8. Why have the controls like age and number of living children been converted to dummy variables for different age categories. It would be good to see the results with age and number of living children as a continuous variable. Similarly years of education is available for both the woman and the man. The authors could include years of education as a continuous variable in their regression as well.
9. Page 6, typo “This Table *have* two models…”. It should be “This Table *has* two models….”
10. The authors claim in the discussion section that WBPCI is significantly associated with women’s contraceptive use. This is contentious. The result is only significant for the unadjusted model, where the authors do not control for any additional explanatory variables. Once additional variables are included, the effect of WBPCI disappears, thus suggesting that women’s bargaining power is no longer an important determinant of modern contraceptive use once you control for exposure to radio & newspaper, desire for more kids, education, number of existing children, husband’s occupation and so on. The authors need to include this as the discussion rather than suggest that WBPCI does play a significant role in determining the log odds of using modern contraception. There is only one line explaining this result in the discussion at the end while most of the discussion pertains to the unadjusted model without any controls. This could be misleading.
11. Women’s age interpretation can be simplified if a continuous variable for age is utilized (information is available as continuous variable in DHS). This way it is easier to interpret if with age, the capacities of procreation decline and whether the data does in fact suggest this. As I have mentioned before, the authors can change the measurement of some of these variables and estimate an alternative model for sensitivity analysis and check.
12. I would like to see some discussion on what could explain the result that husband’s occupational status is associated with lower odds of using contraception?
13. In the limitations section, the authors mention that  women’s bargaining power may be related to certain unobservable factors that also influence contraceptive use suggesting potential endogeneity of WBPCI. However, the authors are not claiming causality and as such this is not a big concern. The paper is concluding only associations.
14. I have reservations about the policy implications the authors make in the conclusion. The authors mention that “findings postulate that women with high bargaining power are able to use the most appropriate reproductive health care, policymakers need to look at the social policies of status revitalization and women’s empowerment within households with much acuity.” This finding is only accurate in the model with no controls and as such cannot be used as a policy suggestion. Once controls are included in the model, women’s bargaining is no longer significant. In fact, the effects of other variables considered are significant and the policy conclusions needs to be derived from those variables.

Is the work clearly and accurately presented and does it cite the current literature?

Yes

If applicable, is the statistical analysis and its interpretation appropriate?

Partly

Are all the source data underlying the results available to ensure full reproducibility?

Yes

Is the study design appropriate and is the work technically sound?

Yes

Are the conclusions drawn adequately supported by the results?

Partly

Are sufficient details of methods and analysis provided to allow replication by others?

No

Reviewer Expertise:

Applied microeconometrics, empirical development economics, economics of the households, health, gender and education

I confirm that I have read this submission and believe that I have an appropriate level of expertise to confirm that it is of an acceptable scientific standard, however I have significant reservations, as outlined above.

# Reviewer report: Reviewer response for version 2

**DOI:** 10.21956/gatesopenres.14509.r36888

**Published:** 2024-07-22

**Authors:** Abiodun Adanikin

**Affiliations:** 1 Coventry University, Coventry, England, UK

Thank you for requesting my assessment of this manuscript, analysing women's bargaining power and modern contraceptive use in Cameroon. The manuscript was generally well written, but there are some concerns regarding data interpretation. The adjusted WBPCI was not significant, with a value of 0.996 (0.918 – 1.081), and I am curious as to whether the basis for the interpretation and conclusion of the manuscript can solely rely on the unadjusted analysis in this case, as all other variables have been interpreted using adjusted methods. This could potentially indicate bias in the interpretation. Furthermore, the interpretation of the impact of higher education and poor household wealth on modern contraceptive use in Table 3 is incorrect.

Is the work clearly and accurately presented and does it cite the current literature?

Yes

If applicable, is the statistical analysis and its interpretation appropriate?

No

Are all the source data underlying the results available to ensure full reproducibility?

Yes

Is the study design appropriate and is the work technically sound?

Yes

Are the conclusions drawn adequately supported by the results?

No

Are sufficient details of methods and analysis provided to allow replication by others?

Yes

Reviewer Expertise:

maternal and child health epidemiology, social statistics and demography

I confirm that I have read this submission and believe that I have an appropriate level of expertise to state that I do not consider it to be of an acceptable scientific standard, for reasons outlined above.

# Reviewer report: Reviewer response for version 2

**DOI:** 10.21956/gatesopenres.14509.r36886

**Published:** 2024-07-19

**Authors:** Patrick O. Asuming

**Affiliations:** 1 University of Ghana, Accra, Greater Accra Region, Ghana

The paper investigates the effect of women's bargaining power on the use of modern contraceptives in Cameroon. The paper uses data from the 2018 Demographic and Health survey and analysis data for 6546 women who were married or in union. The authors created an index of women's bargaining power from a series of indicators and run a logistic regression model to identify the association between bargaining power and contraceptive use. The authors conclude that woman's bargaining power within the household increases the likelihood of contraceptive use.

 Overall, I think the paper addresses a very important issue. It is generally well written.

 My main concern is that the author's main conclusion is based on the crude model (model without covariates) but not on the full model. It is normally expected that the conclusions should be based on the full model, which in this case suggests that women's bargaining power has no influence on the likelihood of use of modern contraceptive. It is not just that the Odds-Ratio (OR) losses significance. Indeed, the OR declines substantially from the crude model to the full model to the extent that it changes signs. This probably suggests that the bargaining power of the women is probably captured by some of the covariates used. I suggest that the authors modify their conclusion accordingly to be more reflective of the full model.

Is the work clearly and accurately presented and does it cite the current literature?

Yes

If applicable, is the statistical analysis and its interpretation appropriate?

Yes

Are all the source data underlying the results available to ensure full reproducibility?

Yes

Is the study design appropriate and is the work technically sound?

Yes

Are the conclusions drawn adequately supported by the results?

Partly

Are sufficient details of methods and analysis provided to allow replication by others?

Yes

Reviewer Expertise:

Health systems research, development economics, health economics.

I confirm that I have read this submission and believe that I have an appropriate level of expertise to confirm that it is of an acceptable scientific standard, however I have significant reservations, as outlined above.

# Reviewer report: Reviewer response for version 2

**DOI:** 10.21956/gatesopenres.14509.r30703

**Published:** 2021-05-19

**Authors:** David K. Guilkey

**Affiliations:** 1 Carolina Population Center, Department of Economics, University of North Carolina at Chapel Hill, Chapel Hill, NC, USA

No further comments.

Is the work clearly and accurately presented and does it cite the current literature?

Partly

If applicable, is the statistical analysis and its interpretation appropriate?

Partly

Are all the source data underlying the results available to ensure full reproducibility?

Yes

Is the study design appropriate and is the work technically sound?

Partly

Are the conclusions drawn adequately supported by the results?

Partly

Are sufficient details of methods and analysis provided to allow replication by others?

Yes

Reviewer Expertise:

Econometric Methods and program evaluation

I confirm that I have read this submission and believe that I have an appropriate level of expertise to confirm that it is of an acceptable scientific standard.

# Reviewer report: Reviewer response for version 1

**DOI:** 10.21956/gatesopenres.14260.r29213

**Published:** 2020-08-06

**Authors:** David K. Guilkey

**Affiliations:** 1 Carolina Population Center, Department of Economics, University of North Carolina at Chapel Hill, Chapel Hill, NC, USA

This paper uses Demographic and Health Survey (DHS) data for Cameroon from 2011 to estimate a model of the determinants of contraceptive use. The key explanatory variable is an index for the strength of a woman’s bargaining power within couples which is constructed using multiple correspondence analysis. The main conclusion is that woman’s bargaining power has a positive and significant effect on contraceptive use and this result is robust to a selectivity correction used to control for dropping pregnant women from the contraceptive use equation.

 Specific comments:

1. The multiple correspondence analysis (MCA) is not adequately described. For example, I assume that the authors used the first dimension from the MCA to construct their index but this is not clearly stated. It would also be useful to know what percent of the variation is explained by this first dimension.
2. It would be nice to know the breakdown of the methods that are included in the 22.9% contraceptive use. In particular, what is the percentage of traditional use? If it is large enough, it would be useful to see a multinomial logit model with three categories: non use, modern use, and traditional use since it may be the case that woman’s bargaining power could have different effects for modern versus traditional use.
3. The authors do not clearly state whether or not their model demonstrates causality or association. It is likely that their index could be considered endogenous to contraceptive use. It is also possible that exposure to radio and television as well as the desire for no more children are endogenous. If these possibilities are true, then the model is one of association.
4. It is not clear to me that the Heckman selection model is a useful addition. First of all, the identifying variable they use as an exclusion restriction from the contraceptive use equation is ideal family size and they state that this variable is exogenous. This is almost certainly not the case and it has been found to be a direct determinant of contraceptive use in other empirical work (see, for example, Cronin *et al.*, 2018 ^1^). Second, selection models tend to be unstable and not robust to minor variations in the model specification.
5. Therefore, I would recommend dropping the selection analysis and then switching to multinomial logit for the determinants of contraceptive use.

Is the work clearly and accurately presented and does it cite the current literature?

Partly

If applicable, is the statistical analysis and its interpretation appropriate?

Partly

Are all the source data underlying the results available to ensure full reproducibility?

Yes

Is the study design appropriate and is the work technically sound?

Partly

Are the conclusions drawn adequately supported by the results?

Partly

Are sufficient details of methods and analysis provided to allow replication by others?

Yes

Reviewer Expertise:

Econometric Methods and program evaluation

I confirm that I have read this submission and believe that I have an appropriate level of expertise to confirm that it is of an acceptable scientific standard, however I have significant reservations, as outlined above.

## References

[1] : The effects of health facility access and quality on family planning decisions in urban Senegal. *Health Econ* .27(3) : 10.1002/hec.3615 576-591 10.1002/hec.3615 29094775 PMC5867202

# Reviewer report: Reviewer response for version 1

**DOI:** 10.21956/gatesopenres.14260.r29255

**Published:** 2020-07-31

**Authors:** Aurélia Lépine

**Affiliations:** 1 Institute for Global Health, University College London, London, UK

This paper has several issues that would need to addressed before indexing:

1. The authors infers causality in the abstract and in other sections of the paper (method, result, discussion) between women's bargaining power and contraceptive use while the analysis cannot say anything about a causal relationship.
2. There is not enough information on the MCA and on the variables included in the women's bargaining power index. Also authors do not discuss potential issues in relying on self-report. In this context I see two main issues 1) social desirability bias and 2) the fact that women who have disproportionate power in the household in context in which this clashes with social norms may actually be more a sign of woman's neglection by her husband or conflict between spouses rather than women's bargaining power. 
3. The sample excluded pregnant women but it is not clear if it excluded women who were not sexually active. In addition, given that it seems that several measures of women's power captured decision-making in the couple it was not clear why the analysis did not focus on married women or women living with their partner. 
4. The dependent variable should have been coded 0 if the woman was using a traditional contraceptive method. 
5. Bivariate analysis does not add anything to the multivariate analysis result and should be removed. In fact, the relationship between women's bargaining power and contraceptive is driven by confounding factors. 

Is the work clearly and accurately presented and does it cite the current literature?

Yes

If applicable, is the statistical analysis and its interpretation appropriate?

No

Are all the source data underlying the results available to ensure full reproducibility?

Yes

Is the study design appropriate and is the work technically sound?

No

Are the conclusions drawn adequately supported by the results?

Partly

Are sufficient details of methods and analysis provided to allow replication by others?

No

Reviewer Expertise:

Health economics.

I confirm that I have read this submission and believe that I have an appropriate level of expertise to state that I do not consider it to be of an acceptable scientific standard, for reasons outlined above.

# Reviewer report: Reviewer response for version 1

**DOI:** 10.21956/gatesopenres.14260.r28708

**Published:** 2020-04-27

**Authors:** Vissého Adjiwanou

**Affiliations:** 1 Department of Sociology, Université du Québec à Montréal (UQAM), Montréal, QC, Canada

This study is important to show the importance of women's decision-making autonomy over their use of contraception, which still remains at fairly low levels in Cameroon. The analyses are well-conducted with particular attention to possible problems of sample selection bias. This study has been conducted several times in different contexts and this study does not innovate enough in this direction.

 That said, there are some corrections to be made to the article.

1. The construction of the independent variable: the authors used the "multiple correspondence analysis" approach to build their autonomy indicator. Then, they subtracted the minimum of the indicator values ​​to have positive values. The problem with this construction is that it is difficult to interpret it as such, and the difficulty of comparing it between countries (which obviously is not the case here). The change of axis is to allow a good interpretation of the intercept in a regression, otherwise, I don't see the importance of this change.
2. The argument of the threshold to use to cut the indicator is a problem, but one can easily proceed as in the construction of poverty quintiles by dividing the indicator into 5 parts (like in the dhs). It can still be used as a quantitative variable in regressions.
3. Too much emphasis is placed on maternal mortality, we have to put this into perspective a little and look at the primary role of contraception which is to limit fertility level of for birth spacing.
4. The authors limited their analysis to couples. I do not see the relevance of this limitation here especially since the partner information that is used is the same that is available for married women with a larger sample. It is better to justify the choice of the sample, otherwise, it is more important to work on all married women I think.
5. In terms of results, the bivariate analysis is not properly conducted. It reversed the place of the dependent and independent variable. For example, in Figure 1, the authors show that the autonomy index is higher for women who use contraception than for other women who don’t. The opposite reasoning is the way to go; that contraceptive use is higher among women who have a high index. But the problem is precisely that with a categorical dependent variable and a continuous independent variable, there is no (simple) way to make a bivariate description.
6. The results presented in Table 3 are therefore poorly presented. To find out, for example, whether a woman's level of education is associated with the use of contraception, the frequencies must be presented in row and not in the column as shown in the table.
7. The Heckman model uses an exogenous variable. In this study, the exogenous variable used is “ideal family size of the woman”. A condition of the exogenous variable is that it does not explain the dependent variable of the second equation (here contraception). However, it is obvious that this variable influences the use of contraception. The ideal number of children is an important variable in the woman's decision to use contraception.

Is the work clearly and accurately presented and does it cite the current literature?

Yes

If applicable, is the statistical analysis and its interpretation appropriate?

Partly

Are all the source data underlying the results available to ensure full reproducibility?

Yes

Is the study design appropriate and is the work technically sound?

Partly

Are the conclusions drawn adequately supported by the results?

Yes

Are sufficient details of methods and analysis provided to allow replication by others?

Yes

Reviewer Expertise:

Demography, quantitative methods, sub-Saharan Africa

I confirm that I have read this submission and believe that I have an appropriate level of expertise to confirm that it is of an acceptable scientific standard, however I have significant reservations, as outlined above.

# Response

**Published:** 2021-09-23

**Authors:** Dimitri TCHAKOUNTE

**Affiliations:** University of Yaounde II, Cameroon

**Responses on the report of Vissého Adjiwanou, **Department of Sociology, Université du Québec à Montréal (UQAM), Montréal, QC, Canada.

*The corrections suggested by the reviewer are very useful. We have thus introduced in the revised paper most of his corrections.*

**Comment 1:** I agree with this comment since in the new version, the values of the Woman Bargaining Power Composite Index (WBPCI) are now comprising between -2.79 and 1.586.

**Comment 2**: We do not affirm that an index should not be categorized (as you explained, the DHS program use such a method), but we refer on the argument suggested by Richardson (2018) to justified why our Index has not been categorized.   

**Comment 3:** As the reviewer suggested, the emphasis has been placed on the primary role of contraception rather than maternal mortality.

**Comment 4:** We follow the instruction of the reviewer concerning this comment. That is why in the revised paper, the study considers only married women or women living in a union with a partner.

**Comment 5:** The reviewer is right. We removed the bivariate analysis in the revised paper.

**Comment 6:** Effectively, ideal family size of the woman seems to be an inadequate exogenous variable. We thus removed this analysis from the revised paper as also suggested by David K. Guilkey.
